# Dried urine spots as sampling technique for multi-mycotoxin analysis in human urine

**DOI:** 10.1007/s12550-021-00423-1

**Published:** 2021-02-26

**Authors:** Jessica Schmidt, Viktoria Lindemann, Monica Olsen, Benedikt Cramer, Hans-Ulrich Humpf

**Affiliations:** 1 Institute of Food Chemistry, Westfälische Wilhelms-Universität Münster, Corrensstr. 45, 48149 Münster, Germany; 2 Risk Benefit Assessment Department, Swedish Food Agency, PO Box 622, 75126 Uppsala, Sweden

**Keywords:** Biomonitoring, Dried urine spot, Mycotoxin, Mass spectrometry, HPLC-MS/MS, Metabolite

## Abstract

A simple and effective approach for HPLC-MS/MS based multi-mycotoxin analysis in human urine samples was developed by application of dried urine spots (DUS) as alternative on-site sampling strategy. The newly developed method enables the detection and quantitation of 14 relevant mycotoxins and mycotoxin metabolites, including citrinin (CIT), dihydrocitrinone (DH-CIT), deoxynivalenol (DON), fumonisin B_1_ (FB_1_), T-2 Toxin (T-2), HT-2 Toxin (HT-2), ochratoxin A (OTA), 2′*R*-ochratoxin A (2′*R*-OTA), ochratoxin α (OTα), tenuazonic acid and *allo*-tenuazonic acid (TeA + *allo*-TeA), zearalenone (ZEN), zearalanone (ZAN), α-zearalenol (α-ZEL), and β-zearalenol (β-ZEL). Besides the spotting procedure, sample preparation includes enzymatic cleavage of glucuronic acid conjugates and stable isotope dilution analysis. Method validation revealed low limits of detection in the range of pg/mL urine and excellent apparent recovery rates for most analytes. Stability investigation of DUS displayed no or only slight decrease of the analyte concentration over a period of 28 days at room temperature. The new method was applied to the analysis of a set of urine samples (*n* = 91) from a Swedish cohort. The four analytes, DH-CIT, DON, OTA, and TeA + *allo*-TeA, could be detected and quantified in amounts ranging from 0.06 to 0.97 ng/mL, 3.03 to 136 ng/mL, 0.013 to 0.434 ng/mL and from 0.36 to 47 ng/mL in 38.5%, 70.3%, 68.1%, and 94.5% of the samples, respectively. Additional analysis of these urine samples with an established dilute and shoot (DaS) approach displayed a high consistency of the results obtained with both methods. However, due to higher sensitivity, a larger number of positive samples were observed using the DUS method consequently providing a suitable approach for human biomonitoring of mycotoxin exposure.

**Supplementary Information:**

The online version contains supplementary material available at 10.1007/s12550-021-00423-1.

## Introduction

Mycotoxins are toxic secondary metabolites of mould fungi. Mould infestation and formation of mycotoxins is influenced by climatic factors like temperature and humidity and can occur at all stages of the production process as well as during storage (Vidal et al. 2018). Biosynthetic origins, chemical structures and biological effects are manifold, and mycotoxin exposure may affect human health due to different toxic effects like acute toxicity, carcinogenicity, teratogenicity, mutagenicity, neurotoxicity, immunosuppression or estrogenic effects (Bennett and Klich 2003). In order to assess the exposure to these contaminants, human biomonitoring proved to be a useful approach. Via the detection of validated mycotoxin biomarkers like the parent toxins themselves and/or metabolised forms in physiological samples, exposure assessment is enabled at the individual level while reducing uncertainties arising from varying degrees of contamination and consumption (Escrivá et al. 2017). For estimation of recent mycotoxin intake, urine is usually the biological matrix of choice while blood, serum or plasma samples have been found more suitable for prolonged exposure assessment (Warth et al. 2013). Biomarker-based methods show distinct advantages, but low analyte concentrations, complex matrices and the broad spectrum of analytes are still challenging (Warth et al. 2012, 2013). In case of urine samples, common procedures of sample clean up like solid-phase extraction or immunoaffinity chromatography may increase sensitivity and specificity, but are time- and cost-intensive and therefore show limited suitability for the analysis of large sample cohorts (Ediage et al. 2012; Solfrizzo et al. 2011; Warth et al. 2013). On that account, dilute and shoot (DaS) approaches are a viable alternative for multi-mycotoxin analysis allowing short analysis times and a high sample throughput while higher limits of detection are acceptable (Gerding et al. 2014; Warth et al. 2012, 2013). Due to the limited availability of mycotoxin glucuronide standards and the potential increasing concentration of the respective parent compounds or phase I metabolites, enzymatic hydrolysis can be a valuable tool of sample pre-treatment (Huybrechts et al. 2015; Muñoz et al. 2017; Šarkanj et al. 2018; Solfrizzo et al. 2011).

Regardless of the sample preparation, liquid urine samples are necessary for the approaches mentioned above, resulting in biological hazards through sample leakage and high expenses for safe and timely shipping of the frozen samples (Zimmer et al. 2013). For this reason, shipping costs can be rather high and sometimes even exceed the costs of the analytical method itself. Dried matrix spots (DMS) obtained by application of physiological samples onto filter paper are a suitable alternative (Sadones et al. 2014). In contrast to liquid samples, dried physiological samples can be easily shipped by mail with simple containments of sturdy paper. The risk of accidental contact to potential infectious material is reduced compared with the liquid samples, and the DMS can be stored at ambient temperature (Resano et al. 2018; Zimmer et al. 2013). Since separation of interfering matrix components on the filter paper may also be possible, DMS can also be taken into consideration as simple and cost-effective alternative of sample preparation (Sadones et al. 2014). Dried urine spots (DUS) have been successfully used for various analytical purposes. Already in 1959, a method using urine dried on filter paper was described for testing phenyl pyruvic acid and further components in order to detect phenylketonuria (Berry 1959). During the last years, several studies determining glycosaminoglycans in urine for diagnosis of mucopolysaccharidosis using DUS have been conducted (Auray-Blais et al. 2012; Breier et al. 2014). Further compounds successfully being analysed in DUS include hippuric acid and creatinine (Antunes et al. 2013) as well as the bile acid profile for detection of inborn errors of bile acid synthesis (Naritaka et al. 2019). Also, in the scope of drug screening, several approaches using dried urine samples have been developed (Lee et al. 2013; Michely et al. 2017, 2018; Otero-Fernández et al. 2013). Because some drugs are almost entirely metabolised into their conjugated analogues, Michely et al. (2017) additionally implemented an on-spot conjugate cleavage in order to obtain higher concentrations of the corresponding phase I metabolites.

After the successful application of dried blood and serum spots (DBS/DSS) for ochratoxin A (OTA) and multi-mycotoxin analysis (Cramer et al. 2015; Osteresch et al. 2017), the aim of this study was to develop and validate a multi-method for the analysis of mycotoxins and mycotoxin metabolites in urine samples by use of DUS. In total, 14 analytes, namely citrinin (CIT) and its metabolite dihydrocitrinone (DH-CIT), deoxynivalenol (DON), fumonisin B_1_ (FB_1_), T-2 Toxin (T-2), HT-2 Toxin (HT-2), OTA, its thermal degradation product 2′*R*-ochratoxin A (2′*R*-OTA), ochratoxin α (OTα), tenuazonic acid and its epimer *allo*-tenuazonic acid (TeA + *allo*-TeA), zearalenone (ZEN), zearalanone (ZAN) and their urinary metabolites α-zearalenol (α-ZEL) and β-zearalenol (β-ZEL), were implemented (Bennett and Klich 2003; Cramer et al. 2008). The spectrum of analytes was chosen based on the expected occurrence in urine samples, toxicity and availability of stable isotope-labelled standards.

## Materials and methods

### Chemicals and reagents

Acetonitrile and methanol used in this study were purchased in LC-MS-grade quality from Fisher Scientific (Schwerte, Germany). A Purelab Flex 2 system (Veolia Water Technologies, Celle, Germany) was used to produce ultrapure water (ASTM type 1 grade). Formic acid was obtained from Merck (Darmstadt, Germany). Whatman qualitative filter paper for technical use, grade 2294 (thickness 1500 µm, pore size 8–15 µm, particle retention 15 µm), ammonium bicarbonate and ammonium acetate were purchased from Sigma Aldrich (Taufkirchen, Germany). β-Glucuronidase was obtained from Megazyme (Bray, Ireland, *E. coli* EC 3.2.1.31, CAZy Family: GH2) or from Sigma Aldrich (*E. coli* Type IX-A and *H. pomatia* Type HP-2). CIT, ZAN and 4-methylumbelliferyl-β-d-glucuronide were from Sigma Aldrich. DH-CIT was purchased from AnalytiCon Discovery (Potsdam, Germany). DON, FB_1_, OTA, T-2, HT-2 and ZEN were isolated and purified from fungal cultures (Beyer et al. 2009; Bittner et al. 2013; Bretz et al. 2006; Cramer et al. 2007, 2010; Hübner et al. 2012). 2′*R*-OTA was produced by thermal isomerisation of OTA (Cramer et al. 2008). TeA, OTα, *d*_5_-OTA and *d*_5_-2′*R*-OTA were synthesised chemically by Lohrey et al. (2013) and Cramer et al. (2010). α-ZEL and β-ZEL were obtained by reduction of ZEN according to Urry et al. (1966). The glucuronic acid-conjugated metabolites DON-3-GlcA, HT-2-3-GlcA, HT-2-4-GlcA, ZEN-14-GlcA, α-ZEL-14-GlcA and β-ZEL-14-GlcA were produced enzymatically by use of rat and pig liver microsomes (Welsch and Humpf 2012). The stable isotope-labelled standards, ^13^C_3_-CIT, ^13^C_3_-DH-CIT, ^13^C_2_-OTα and ^13^C_2_-TeA, were synthesised chemically (Bergmann et al. 2018; Beyer et al. 2009; Bretz et al. 2006; Cramer et al. 2007, 2010; Lohrey et al. 2013). Labelling of FB_1_ was achieved by acid-catalysed oxygen exchange from H_2_^18^O, and a mixture of different isotopologues was obtained (Bergmann et al. 2013). The synthesis of *d*_1_-DON was based on its natural precursor 3-acetyldeoxynivalenol (Bretz et al. 2006). The respective unlabelled mycotoxins were used for the synthesis of *d*_9_-HT-2 (unpublished data), *d*_3_-T-2 and *d*_2_-ZEN (Beyer et al. 2009; Cramer et al. 2007).

The exact concentrations of the majority of in-house produced standards were determined by UV spectroscopy. The concentrations of the stock solutions of DON, FB_1_, T-2 and HT-2 however were determined by quantitative nuclear magnetic resonance spectroscopy (qNMR) using an internal calibration with Thymol (Sigma Aldrich, Taufkirchen, Germany). ^1^H-NMR spectra were recorded on a 600 MHz Agilent DD2 600 MHz spectrometer (Agilent, Waldbronn, Germany) using an adapted recording method for the quantitative determination (Pauli et al. 2015). Spectra processing and calculations were carried out using MestReNova (software version: 12.0.3-21384, Mestrelab Research S.L., Santiago de Compostela, Spain) respectively Excel 2016 (Microsoft Corporation, Redmond, USA). Two working solutions containing all analytes were prepared in ACN/H_2_O (50/50, *v*/*v*) at 10- or 100-fold concentration of the highest calibration point. For the stable isotope-labelled standards, two working solutions were prepared at 10- and 100-fold concentration of their respective concentrations in the injection solutions. All working solutions were stored at −18 °C.

### Sample collection

To obtain urine containing only negligible amounts of mycotoxins and mycotoxin metabolites, three female volunteers avoided the consumption of cereal-based foodstuffs for 36 h prior to the urine donation. The samples were analysed and except for OTA and TeA + *allo*-TeA, which were only determined in traces below the respective limits of quantitation (LOQs) (OTA 0.013 ng/mL urine and TeA 0.36 ng/mL urine, see Table 1), none of the analytes of interest was detectable. For compensation of differences in individual urine compositions, the three urine samples were combined and used as blank urine samples for matrix-matched calibrations, spiking experiments and quality controls. The new method was applied to the analysis of urine samples from adolescents in Sweden, which were collected as part of a previously published study (Warensjö Lemming et al. 2020). All urine samples were stored at −18 °C until analysis. Ethical approval was received from the Regional Ethical Review Board in Uppsala (No. 2015/190) and participants or rather the legal guardian(s) for children younger than 16 years gave written consent prior to urine donation.

**Table 1:** Validation parameters of the developed dried urine spot (DUS) method

Analyte	LOD (ng/mL urine)	LOQ (ng/mL urine)	Working range (ng/mL urine)	Regression type	Regression coefficient (*R*^2^)	EE (%) *n* = 3	R_A_ (%) *n* = 3	Intraday repeatability (%) *n* = 12	Interday repeatability (%) *n* = 4 (3 days)
CIT	0.06	0.20	0.18–18	Linear	0.9995	59.7	56.5	7.1	8.4
DH-CIT	0.02	0.06	0.06–6.0	Linear	0.9996	97.1	96.7	8.4	7.9
DON	0.91	3.03	3.0–300	Linear	0.9989	95.4	93.9	6.5	5.9
FB_1_	0.16	0.53	0.54–54	Linear	0.9972	100.9	111.3	11.3	20.0
T-2	0.10	0.33	0.30–30	Linear	0.9997	95.3	93.4	7.4	8.7
HT-2	1.4	4.5	4.5–225	Linear	0.9977	91.9	101.1	16.3	24.1
OTA	0.004	0.013	0.012–1.2	Linear	0.9972	84.9	87.7	11.2	15.0
2′*R*-OTA	0.005	0.016	0.012–1.2	Linear	0.9998	84.4	90.0	9.6	12.1
OTα	0.04	0.14	0.12–12	Linear	0.9995	94.8	90.8	8.5	4.8
TeA + *allo*-TeA	0.11	0.36	0.36–36	Linear	0.9995	77.4	83.7	7.4	8.7
ZAN	0.17	0.57	0.60–60	Quadratic	0.9965	86.8	81.0	17.2	24.9
ZEN	0.09	0.31	0.30–30	Quadratic	0.9948	27.3	24.8	37.9	66.1
α-ZEL	0.17	0.58	0.60–60	Quadratic	0.9937	22.9	20.9	12.8	43.8
β-ZEL	0.26	0.85	0.84–84	Quadratic	0.9990	26.2	11.5	15.0	46.5

*LOD* limit of detection, defined at a S/N ratio of 3, *LOQ* limit of quantitation, defined at a S/N ratio of 10, *EE* extraction efficiency, *R*_*A*_ apparent recovery

### Sample preparation

Urine samples were allowed to reach room temperature, vortexed vigorously and centrifuged for 5 min at 15,000×*g*. For enzymatic hydrolysis of the mycotoxin glucuronides, 55 µL ammonium bicarbonate buffer (1 M, pH 6.8) containing 3300 U of β-glucuronidase from *E. coli* (Megazyme, EC 3.2.1.31, CAZy Family: GH2) was added to 550 µL of the supernatant and incubated for 16 h at 37 °C with continuous shaking at 120 rpm (Rotamax 120, Heidolph Instruments GmbH & Co. KG, Schwabach, Germany). Five hundred fifty microliters of the digested sample was pipetted on the lower edge of a filter paper rectangle (3 × 4 cm). After drying at room temperature overnight, the upper part of the filter paper, which contained no urine, was removed. The dried urine spot (DUS) was transferred to a 5-mL safe-lock tube and extracted with 3 mL of the extraction solvent comprising ACN, MeOH and H_2_O (35/35/30, *v*/*v*/*v*) for 45 min under sonication. Subsequently, an aliquot of 1 mL was pipetted into a new 1.5-mL safe-lock tube before the addition of 10 µL of each of the two working solutions of the isotope-labelled standards. Following evaporation to dryness at 45 °C under reduced pressure, the residues were reconstituted with 100 µL of mobile phase comprising the initial solvent composition of the HPLC separation (H_2_O/ACN/formic acid, 95/5/0.1, *v*/*v*/*v*). The solution was centrifuged for 5 min at 15,000×*g*, and the supernatant subjected to HPLC-MS/MS analysis. All samples were analysed in duplicate in batches containing at least one medium concentration quality control standard every twenty samples. Furthermore, a urine sample endowed with 3 ng/mL of 4-methylumbelliferyl-β-d-glucuronide was incubated separately as positive control to monitor the β-glucuronidase activity (Prasain 2012). Determination of creatinine levels was carried out according to Mazzachi et al. (2000).

In terms of optimising the enzymatic hydrolysis blank urine was spiked with DON-3-GlcA, HT-2-3-GlcA, HT-2-4-GlcA, ZEN-14-GlcA, α-ZEL-14-GlcA and β-ZEL-14-GlcA and different protocols were applied. Besides the above-mentioned procedure, an aqueous solution of β-glucuronidase from *H. pomatia* was diluted with 2.5 M ammonium acetate buffer at pH 4.5 to obtain an enzyme concentration of 60.000 U/mL. Additionally, diluted solutions of the two β-glucuronidase-buffer mixtures containing 600 U/mL at buffer concentrations of 0.2 M ammonium acetate or ammonium bicarbonate at constant pH values were used. Applying the low concentrated enzyme-buffer solutions, 550 µL of the mixture was added to 550 µL of the urine sample resulting in a dilution by factor 1.8 compared with the standard protocol described above.

### HPLC–MS/MS conditions

A QTRAP 6500 mass spectrometer (SCIEX, Darmstadt, Germany) coupled to a 1260 Infinity LC system (Agilent, Waldbronn, Germany) was used for analysis. Analytes of interest were separated on a Nucleodur C_18_ Pyramid column (3 µm, 2.0 × 100 mm) equipped with a guard column of the same material (2.0 × 4.0 mm) (both Macherey-Nagel, Düren, Germany). A binary gradient consisting of ACN (A) and H_2_O (B), both containing 0.1% formic acid, was applied at a flow rate of 600 µL/min. At a column temperature of 45 °C, the HPLC starting conditions of 5% A were held constant for 2 min, followed by a linear increase of solvent A to 95% at 9.5 min. The conditions were maintained for 0.5 min. Afterwards, the percentage of A was decreased to starting conditions until 10.1 min followed by re-equilibrating the column for 2.9 min resulting in a total runtime of 13 min. The injection volume was set to 30 µL. In order to avoid unnecessary contamination of the mass spectrometer with salts and other matrix components, the first 2 min of every chromatographic run was directed into the waste port.

Ionisation was achieved by electrospray ionisation (ESI) using the scheduled multiple reaction monitoring (sMRM) detection mode, which monitors the MRM transitions of each analyte only in a predefined window of retention. Ion spray voltage was set to 5500 V in positive and −4500 V in negative ionisation mode at a source temperature of 500 °C. The curtain gas was set to 40 psi, and the ion source gases 1 and 2 were set to 45 psi and 55 psi, respectively. The analyte-dependent MS/MS parameters, including the declustering potential (DP) and collision energy (CE), were optimised and two individual MRM transitions monitored for each compound. The scheduled MRM parameters were set to a window width of at least 30 s and a target scan time of 0.1 s. Data acquisition was performed using Analyst 1.6.2 software, and Multiquant 3.0.3 was used for data processing. Detailed MS-parameters for all analytes are listed in Tables S1 and S2 (Electronic Supplementary Material).

### Validation experiments and stability testing

In order to evaluate method performance, an in-house validation was carried out including linearity, limits of detection (LODs), limits of quantitation (LOQs), extraction efficiency (recovery) and apparent recovery (absolute recovery) as well as intra- and interday repeatability. The results of the validation experiments are summarised in Table 1.

The LOD and LOQ were determined based on matrix-matched calibrations. To that end, extracts of blank urine samples were spiked at nine concentration levels in a range of one magnitude around the estimated values. The calculation of LOD and LOQ was based on a linear regression of the S/N ratio and the respective concentrations of five of the nine equidistant calibration points. A S/N ratio of 3 to calculate LOD and a S/N ratio of 10 for calculating LOQ were applied. For determination of further validation parameters, mixtures of the analytes and the corresponding stable isotope-labelled internal standards, which were available for most analytes, were used. Thus *d*_2_-ZEN was chosen as internal standard for ZEN, ZAN, α-ZEL and β-ZEL. The final working range consisted of nine calibration points covering two decimal powers with constant concentrations of the respective internal standards (see Table S3, Electronic Supplementary Material). Only for HT-2, the calibration range was limited from 4.5 to 225 ng/mL.

In assessing which regression model fits best for each analyte, different aspects were considered. The coefficients of variation were calculated for a linear and a quadratic regression model, and Mandel’s fitting test was applied (Mandel 1964). For all analytes using *d*_2_-ZEN as internal standard, the quadratic regression model was preferred to the linear regression model. The deviation from the linear model was caused by an interference with the naturally occurring ^13^C_2_ isotopomer of ZEN (Cramer et al. 2007). For all other analytes, a linear fit was confirmed, and the coefficients of determination (*R*^2^) were considered as additional criterion for linearity of the calibration curves.

The assessment of extraction efficiencies (EEs) and apparent recoveries (R_A_) required three different types of spiking experiments. Besides simple solvent-based calibrations, matrix-matched calibrations were prepared by addition of the analyte standards after the extraction step. Since the mycotoxin glucuronides were cleaved in the cause of the enzymatic hydrolysis, data evaluation was solely based on the parental compounds. To obtain matrix calibrations, blank urine was therefore spiked with the parental compounds of all analytes followed by enzymatic hydrolysis and spotting. The internal standard solutions were added after the extraction step. All calibrations types were prepared in triplicate at nine concentration levels each. Based on the slopes of the linear calibrations, EE and R_A_ were calculated according to the following equations, taking the internal standards into account (modified by the equations of Matuszewski et al. (2003)):

$$EE\left(\%\right)=\frac{{slope}_{matrix\;calibration}}{{slope}_{matrix-matched\; calibration}}\times 100$$

$${R}_{A}\left(\%\right)=\frac{{slope}_{matrix\; calibration}}{{slope}_{solvent\;calibration}}\times 100$$

In the case of a quadratic calibration function, instead of the slope, the sensitivity was used for calculations. The sensitivity depends on the respective concentration value and corresponds to the first derivative of the calibration function. To calculate the process parameters, the sensitivity should be specified in the middle of the working range (Funk 2005).

As a measure of precision of the developed method, the relative standard deviation during intra- and interday repeatability was evaluated. To investigate intraday repeatability, the pooled blank urine sample was spiked with the parental compounds of all analytes at a medium concentration level, and sample preparation as well as HPLC-MS/MS analysis was carried out 12 times. Interday repeatability was assessed by sample preparation and measurement in quadruplicate on three different days using the same spiked urine as for the determination of intraday repeatability.

**Fig. 1:**
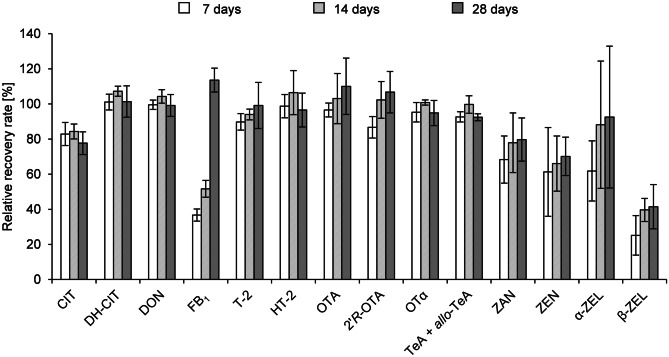
Relative analyte recovery rates after blank urine was spiked at medium concentration levels and stored as dried urine spots for 7, 14 and 28 days at 22 °C; *n* = 4 for each storage time

In the course of stability tests, blank urine samples at medium spiking level were hydrolysed enzymatically and spotted onto filter paper. After drying overnight at room temperature, the samples were packed in plastic bags and stored at 22, 4 and −18 °C for 7, 14 and 28 days before analysis. A fourfold determination was carried out for each combination of storage period and temperature. Recovery rates of the analytes in the stored samples were calculated via matrix-matched calibrations and referred to freshly prepared recovery samples in order to assess the percentage of analyte reduction (see Fig. 1 and Table S4, Electronic Supplementary Material). Analysed urine samples were not corrected for recovery.

### Comparison of dried urine spots and a dilute and shoot approach

The urine samples analysed in this study had previously been analysed as part of a human biomonitoring survey using a dilute and shoot (DaS) approach for determination of mycotoxin concentrations in urine samples (Warensjö Lemming et al. 2020). Concisely, urine samples were thawed, vortexed vigorously and centrifuged for 5 min at 15,000×*g*. A volume of 11.1 µL of the supernatant was diluted with 100 µL of H_2_O/ACN/FA (95/5/0.5; *v*/*v*/*v*) and subjected to HPLC-MS/MS analysis according to Gerding et al. (2014).

## Results and discussion

### Method development and sample preparation

A new sample preparation protocol for mycotoxin analysis in urine samples has been developed. Method development was motivated by enabling a separation between on-site sample preparation and sample analysis in a dedicated HPLC-MS laboratory on the one hand and improving the sensitivity of human biomonitoring on the other hand. The newly developed approach allows the analysis of 14 mycotoxins and mycotoxin phase I metabolites, namely CIT, DH-CIT, DON, FB_1_, T-2, HT-2, OTA, 2′*R*-OTA, OTα, TeA + *allo*-TeA, ZAN, ZEN, α-ZEL and β-ZEL as well as their glucuronides after enzymatic hydrolysis. These compounds were selected based on their toxicological relevance, the reported occurrence rates and the availability of stable isotope-labelled standards.

As a first step of sample preparation, an enzymatic hydrolysis of glucuronic acid metabolites was implemented in order to reduce the number of metabolites accounting for every mycotoxin and to allow a quantitation via stable isotope dilution analysis. This approach results in an increase of the concentration of the phase I metabolites or the non-metabolised parent compounds and thus in an improved overall sensitivity. Subsequent to hydrolysis, an aliquot of the hydrolysed urine was applied to a filter paper strip and dried overnight. The obtained solid urine residue (dried urine spot, DUS) can be prepared in each laboratory and easily shipped by mail with simply containments of sturdy paper, depending on local regulations. This part of the sample preparation is intended to be done in remote laboratories without HPLC-MS/MS facility. Subsequent sample preparation consists of extraction of the DUS followed by HPLC-MS/MS analysis, which should be done in the receiving HPLC-MS/MS laboratory.

In the course of optimising the hydrolysis protocol, β-glucuronidase from different sources (*E. coli* and *H. pomatia*) was tested in terms of complete cleavage of relevant glucuronic acid metabolites (DON-3-GlcA, HT-2-3-GlcA, HT-2-4-GlcA, ZEN-14-GlcA, α-ZEL-14-GlcA and β-ZEL-14-GlcA). Molluskan β-glucuronidase offers the advantage to possess a significant sulfatase activity besides the ability to hydrolyse glucuronides, while glucuronidase from *E. coli* selectively hydrolyses glucuronides without reported side activities. Aqueous solutions of β-glucuronidase from *H. pomatia* were prepared by dilution with ammonium acetate buffer at pH 4.5 whereas for *E. coli*, ammonium bicarbonate buffer at pH 6.8 was used. Buffers and pH values were chosen under consideration of maximal glucuronidase activity. After treatment of a urine sample, which was fortified with the mycotoxin glucuronides mentioned above, with β-glucuronidase from *H. pomatia*, DON-3-GlcA and HT-2-3-GlcA were still detectable. Using β-glucuronidase from *E. coli*, a complete cleavage of all glucuronides was observed while the signal of the respective parent compounds increased correspondingly. Thus, despite leaving potential mycotoxin sulfate conjugates uncleaved, β-glucuronidase from *E. coli* was favoured due to the complete cleavage of the mycotoxin glucuronides.

To obtain a high analyte concentration on the paper strip, efforts were made to minimise the dilution of the urine sample during enzymatic hydrolysis. For that purpose, a high concentrated buffer-enzyme solution resulting in a lower volume added to the urine sample was preferred in comparison with a less concentrated solution. Finally, 55 µL ammonium bicarbonate buffer (1 M, pH 6.8) containing 3300 U β-glucuronidase from *E. coli* was added to 550 µL of urine and incubated at 37 °C for 16 h. The addition of high concentrated buffer allowed a robust adjustment of the pH value independent of the initial pH of the urine sample to assure maximal β-glucuronidase activity.

Reliable quantitation of mycotoxins in urine has been shown to be possible with matrix-matched calibrations when the urine sample is at least tenfold diluted (Gerding et al. 2014; Warth et al. 2012). If this dilution step is not applied, sample-dependent differences in urine concentration and composition strongly affect the signal intensity during the HPLC-MS/MS measurement. To avoid this sample dilution while improving analytical accuracy, stable isotope-labelled standards were added to the urine sample during extraction of the paper strip. Extraction of analytes of interest was performed by adding 3 mL of a mixture consisting of ACN, MeOH and H_2_O (35/35/30; *v*/*v*/*v*). Previously, different mixtures of extraction solvents were tested containing acetone, ACN, MeOH, ethyl acetate, formic acid and H_2_O. However, probably due to the high amount of salts and ionic compounds precipitated on the paper strip, only solvents containing H_2_O were able to extract the polar mycotoxins such as DON sufficiently. Since multi-methods often form a compromise caused by the structural diversity of the compounds, lower extraction rates for some analytes were considered as acceptable, and a mixture of ACN, MeOH and H_2_O (35/35/30; *v*/*v*/*v*) was finally selected for extraction. The influence of the extraction time was investigated over a period of 15 and 75 min, and the highest extraction rates could be achieved after an extraction time of 45 min. Subsequent to this step, 1 mL of the extract was evaporated to dryness followed by reconstitution in the HPLC-MS/MS solvent, centrifugation and injection into the HPLC-MS/MS system. The short analysis time of 13 min per chromatographic run using a simple binary gradient enables a high sample throughput. TeA and its epimer *allo*-TeA were not chromatographically separated as a standard reversed-phase HPLC column was used. For separation of these compounds, additives unsuitable for mass spectrometry, derivatisation or columns with different stationary phases like porous graphitic carbon are required (Hickert et al. 2015, 2017; Hövelmann et al. 2016).

### Validation

Within the scope of the validation, limit of detection (LOD), limit of quantitation (LOQ), extraction efficiency (EE) and overall recovery (R_A_) of the analytes were determined. Furthermore, relative standard deviation during intra- and interday repeatability was assessed as a measure of precision (see “Materials and methods” for further details).

Lowest LODs were achieved for OTA and 2′*R*-OTA with values of 0.004 and 0.005 ng/mL urine, respectively. For DH-CIT, OTα and CIT LODs between 0.02 and 0.06 ng/mL urine were observed. Slightly higher values were determined for ZEN, T-2, TeA + *allo*-TeA, FB_1_, ZAN, α-ZEL and β-ZEL in a range of 0.09 to 0.26 ng/mL urine. The highest LODs were calculated for DON with 0.91 ng/mL urine and HT-2 with 1.4 ng/mL urine (see Table 1). In comparison with the multi-method by Gerding et al. (2014) using a dilute and shoot (DaS) approach without sample clean up, LODs for most analytes were in a similar range of concentration. For DH-CIT and OTA even 10- to 25-fold lower LODs were achieved, while for DON, ZEN and β-ZEL, slightly higher values were obtained. Due to the enzymatic cleavage in the herein developed method, the sum of the unconjugated parent compounds and the respective former glucuronic acid conjugates was detected. This results in a higher expected amount of the parent compounds so that higher LODs compared with methods without hydrolysis can be considered as sufficient. In contrast to the method by Gerding et al. (2014), another DaS method by Warth et al. (2012) provided higher LODs for all analytes than the herein shown results. Other published methods often use a sample cleanup by solid-phase extraction (SPE) or immunoaffinity chromatography columns (IAC) (Šarkanj et al. 2018; Solfrizzo et al. 2011). The separation of interfering matrix compounds and the higher specificity of these approaches partially led to an improved sensitivity in relation to the new DUS method (Huybrechts et al. 2015; Šarkanj et al. 2018). As the aim of this study was to develop a method simplifying storage, transport and handling of urine samples, the slightly higher LODs are regarded as highly satisfying. Despite often reported chromatographic difficulties for the analysis of TeA, excellent values for LOD and LOQ could be achieved.

In order to compensate matrix effects, different calibration strategies can be used, e.g. standard addition, external matrix-matched calibration or the addition of stable isotope-labelled standards. Because a standard addition method multiplies the workload as well as the required sample amount for each sample, this approach is not suitable for high sample throughput. Also, matrix-matched calibrations do not always enable the reliable compensation of matrix effects due to varying composition and concentration of the urine between different individuals. For this reason, stable isotope-labelled standards were used to assure a reliable quantitation.

Extraction efficiency (EE) was calculated by comparing signal intensities of matrix-matched and matrix calibrations as described above. As the stable isotope-labelled standards were added to the aliquot after the extraction step, the matrix calibration does not compensate for losses of compounds of interest before and during the extraction procedure. Despite this limitation, the described procedure was used for further experiments as the motive of this method particularly was to simplify the handling of urine samples. In regions with limited laboratory equipment and without availability of internal standards, enzymatic hydrolysis and spotting can be realised locally while further steps of sample preparation and analysis can be carried out in another more specialised laboratory. Lowest EEs were monitored for ZEN, α-ZEL and β-ZEL with 27.3%, 22.9% and 26.2%, respectively. CIT showed medium EE with 59.7%. For all other analytes, high EEs with percentages of 77.4 to 100.9% were calculated.

The apparent recovery (R_A_) was calculated by dividing the slope of matrix calibrations by the slope of solvent-based calibrations. The respective sensitivity was used to calculate R_A_ for ZEN, ZAN, α-ZEL and β-ZEL as described above, and highly satisfying results were obtained for most analytes. For DH-CIT, DON, FB_1_, T-2, HT-2, OTA, 2′*R*-OTA, OTα, TeA + *allo*-TeA and ZAN R_A_ were monitored in a range of 81.0 to 111.3%. Due to lower extraction efficiency for CIT, R_A_ in a medium range with 56.5% was determined. Low extraction efficiencies for α-ZEL, β-ZEL and ZEN led to R_A_ of 11.5 to 24.8%. For the analysis of mycotoxins in physiological samples, no legal criteria regarding method performance have been established. The obtained values were therefore related to commission regulation (EC) 401/2006 describing the methods of sampling and analysis for the official control of mycotoxin levels in foodstuffs, although the matrices of food samples and physiological samples are rather different (EC 2006). Besides CIT, ZEN, α-ZEL and β-ZEL, apparent recoveries of the other analytes were in line with the therein indicated specifications. The lower values for CIT, ZEN, α-ZEL and β-ZEL were considered as sufficient as they are caused by the low extraction efficiencies. As already discussed above, the selected extraction solvent represents a compromise to cover a broad range of structurally diverse mycotoxins.

In order to evaluate the precision of the developed method, the standard deviation during intra- and interday repeatability was determined. In case of intraday repeatability, a blank urine sample was spiked with all analytes of interest at a medium concentration level, and sample preparation as well as measurement of the samples was carried out 12 times. Interday repeatability was assessed by sample preparation of the same endowed urine samples in quadruplicate on three different days over a period of 5 weeks. Relative standard deviation during intraday repeatability ranged from 6.5 to 17.2% for all analytes, except for ZEN with a higher value of 37.9%. Interday repeatability was assessed to be ≤ 20% for nine analytes, including CIT, DH-CIT, DON, T-2, FB_1_, OTA, 2′*R*-OTA, OTα and TeA + *allo*-TeA. For HT-2 and ZAN, slightly higher values of 24.1% and 24.9%, respectively, were observed. High relative standard deviations during interday repeatability of 43.8 to 66.1% were determined for α-ZEL, β-ZEL and ZEN. Again, the obtained results were classified with respect to commission regulation EC 401/2006 (EC 2006) and only ZEN, α-ZEL and β-ZEL showed higher relative standard deviations due to the low extraction efficiencies as discussed above.

### Stability

Liquid samples have the drawback that the cold chain should not be disrupted during storage and transport. Furthermore, leaks in urine containers can represent a potential biological hazard. The newly developed method is intended to simplify storage and transport of urine samples. To that end, the stability of the analytes of interest in DUS was investigated after storage periods of 7, 14 and 28 days and storage temperatures of 22, 4 and −18 °C (Table S4, Electronic Supplementary Material). Figure 1 shows the time-dependent relative recovery rates of all analytes after storage of the DUS at 22 °C. For DH-CIT, DON, T-2, HT-2, OTA, 2′*R*-OTA, OTα and TeA + *allo*-TeA neither a time- nor a temperature-dependent decrease of the analyte concentration during storage could be observed. Regardless of the storage conditions, relative recovery rates of these analytes were determined to be > 86%. FB_1_ showed the highest remaining toxin concentration in DUS after storing for 28 days at room temperature of more than 100% of the original analyte concentration, whereas comparatively low relative recovery rates of 36.7% and 51.6% were observed after 7 and 14 days, respectively. As a possible explanation, a degradation of certain matrix compounds during storage and subsequent effects on the extractability could be taken into account. Strongest deviations were monitored for ZAN, ZEN, α-ZEL and β-ZEL, but no trend indicating a time- or temperature-dependent reduction was perceived. The high standard deviations of the fourfold determination for each storage time and temperature are in line with low precision during intra- and interday repeatability (see Table 1). Despite the high standard deviations, ZAN and α-ZEL show no to moderate reduction of analyte concentration with remaining percentages of 61.8 to 110.3%. In contrast to this, higher losses were observed for ZEN and β-ZEL with the lowest relative recovery rate of 25.1%. Only for CIT, a slight temperature-dependent decrease was monitored with lowest remaining amounts after storage at 22 °C at each point in time (see Table S4, Electronic Supplement Material). Nevertheless, 77.6% of the original analyte concentration was found after storage at room temperature for 28 days, demonstrating the high stability of this mycotoxin in DUS. Osteresch et al. (2017) investigated the stability of mycotoxins and mycotoxin metabolites in dried blood spots (DBS) and dried serum spots (DSS). With exception of OTA and 2′*R*-OTA, analyte concentration decreased with storage time, when the DBS and DSS were stored at room temperature. The degree of analyte reduction was dependent of the analyte and varied from the remaining relative analyte recovery of 80% for DON to less than 20% for DH-CIT after storage for 28 days. As in DUS neither a clear time nor temperature-dependent decrease of the concentration of the herein investigated mycotoxins could be observed, it can be concluded that DUS can easily be stored at room temperature up to 28 days.

### Biomonitoring of urine samples

The developed DUS method was finally applied for the analysis of 91 urine samples obtained from Swedish adolescents. Sample preparation was carried out in duplicate, and quantitation was performed via matrix-matched calibration containing stable isotope-labelled standards. In total, four out of 14 analytes were detectable. The most frequent occurring mycotoxin was TeA + *allo*-TeA found in 98.9% of the samples with a mean value of 5.9 ng/mL urine, a median of 3.6 ng/mL urine and a highest quantitated level of 47 ng/mL urine. OTA was detectable in 81.2% of the analysed urine samples with mean, median and maximum concentrations of 0.058 ng/mL urine, 0.040 ng/mL urine and 0.434 ng/mL urine, respectively. The occurrence of DON and its hydrolysed metabolites in the analysed sample set was 75.8% with a mean concentration of 21 ng/mL urine, a median of 12 ng/mL urine and a highest calculated concentration of 136 ng/mL urine. For DH-CIT, detectable in 54.9% of the samples, respective mean, median and maximum concentration levels of 0.28 ng/mL urine, 0.17 ng/mL urine and 0.97 ng/mL urine were calculated (see Table 2). The summarised results adjusted for creatinine can be found in Table S5, and detailed results of all individual samples are listed in Table S6 (Electronic Supplementary Material).

**Table 2:** Mycotoxin concentrations in Swedish urine samples determined by application of the newly developed DUS method (*n* = 91)

Mycotoxin	Positive samples % (*n*)	Quantitated samples % (*n*)	Concentration (ng/mL)			
			Mean^a^	Median^a^	Maximum	SD quantitated samples
DH-CIT	54.9 (50)	38.5 (35)	0.28	0.17	0.97	0.25
DON	75.8 (69)	70.3 (64)	21	12	136	26
OTA	81.2 (74)	68.1 (62)	0.058	0.040	0.434	0.064
TeA + *allo*-TeA	98.9 (90)	94.5 (86)	5.9	3.6	47^b^	8.5

*SD* standard deviation

^a^Means and median were calculated from samples with mycotoxin concentrations > LOQ

^b^Values were calculated by extrapolation of the calibration curve

In addition to the new DUS method, the sample set has previously been analysed in the course of a biomonitoring study in Swedish adolescents using an established DaS method (Gerding et al. 2014; Warensjö Lemming et al. 2020). Both approaches of sample preparation were compared in terms of sensitivity and accuracy (Table 3). As the DaS approach is a direct method without enzymatic cleavage, DON and DON-GlcA were monitored separately. To enable the comparison between the two methods, the total DON concentration was calculated from the sum of DON and DON-GlcA. Since TeA was not included in the DaS method, only the obtained values for DH-CIT, DON and OTA were compared. After application of the DaS approach, DH-CIT was found in 15.4% of the urine samples, but in not quantifiable amounts. By use of the DUS approach, 54.9% of the samples were positive for the citrinin metabolite, and 37.4% of the analysed samples were quantifiable. Detectable amounts of DON and/or its glucuronic acid metabolites were observed in 54.9% of the urine samples, and 39.6% could be quantified with the DaS method. The DUS procedure led to a percentage of 75.8% of the urine samples in which DON was detectable. Quantitation of DON was feasible in 70.3% of the analysed urine samples. Using the DaS approach, OTA was only detected in 1.1% of the samples whereas the mycotoxin was detected in 81.3% of the samples by application of the DUS method. The latter led to quantifiable OTA concentrations in 68.1% of the urine samples. Accuracy of the two procedures could only be compared with regard to DON as no other mycotoxin was determined with both methods in quantifiable amounts. The mean calculation of 21 ng/mL DON using the DUS approach and 25 ng/mL by application of the DaS method are highly comparable. Also, median and maximum concentrations obtained with both procedures were in good agreement (see Table 3). Detailed results of all individual samples analysed by application of the DaS approach can be found in Table S7 (Electronic Supplementary Material).

**Table 3:** Mycotoxin concentrations in Swedish urine samples determined by application of the DaS (Warensjö Lemming et al. 2020) and the DUS method (*n* = 91)

	DH-CIT		DON		OTA	
	DUS	DaS	DUS	DaS^b^	DUS	DaS
LOQ (ng/mL)	0.06	0.70	3.0	5.6	0.013	0.030
Positive samples % (*n*)	54.9 (50)	15.4 (14)	75.8 (69)	54.9 (50)	81.3 (74)	1.1 (1)
Quantitated samples % (*n*)	37.4 (34)	0 (0)	70.3 (64)	39.6 (36)	68.1 (62)	0 (0)
Mean^a^ (ng/mL)	0.29	–	21	25	0.058	–
Median^a^ (ng/mL)	0.17	–	12	12	0.040	–
Maximum (ng/mL)	0.97	–	136	139	0.434	–
SD quantitated samples (ng/mL)	0.25	–	26	31	0.064	–

*SD* standard deviation

^a^Means and median were calculated from samples with mycotoxin concentrations > LOQ

^b^Total DON was calculated from the sum of DON and DON-GlcA

The mean concentrations of DON determined in the DUS samples (Table 2) are in good accordance with the results of a German cohort using a DaS approach without enzymatic hydrolysis. In that study, Gerding et al. (2014) observed mean urinary concentrations of 3.38 ng/mL and 15.5 ng/mL for DON and DON-GlcA, respectively. DH-CIT was only monitored below the respective LOQ, which was about tenfold higher than in the herein presented method. OTA was not detectable, and TeA was not included in the method developed by Gerding et al. (2014). Besides exposure assessment in Swedish adolescents leading to the results discussed above, Mitropoulou et al. (2018) investigated mycotoxin occurrence in the urine of Swedish adults and children. The latter study displayed an incidence of DON in 63% and OTA in 51% of the samples obtained from Swedish adults whereas for children, a higher incidence of both mycotoxins was observed with 94% of the samples positive for DON and a percentage of 96% of the samples positive for OTA. Mean urinary concentrations of DON were in a similar range of concentration with 5.37 and 3.89 ng/mL for adults and children, respectively. Minor variation in the concentration levels of OTA between the two age groups was observed, and concentrations were determined to be 0.89 ng/mL urine for adults and 0.18 ng/mL urine for children (Mitropoulou et al. 2018). Although for DON slightly higher levels and lower concentrations of OTA were found in this survey, compared with our study, the determined values are still in the same range of concentration and deviations may occur due to different sizes of the investigated cohorts and the year of sample collection. Regarding the analyte spectrum of the newly developed DUS method, besides DON and OTA, also ZEN and FB_1_ were detected in urine samples of Swedish adults and children. Due to a sample clean up based on SPE and IAC, lower LODs were achieved in the method used by Wallin et al. (2013; 2015) and Mitropoulou et al. (2018) resulting in positive detects for ZEN and FB_1_ in lower concentration levels than accomplished by the DUS.

A sample set from northern Nigeria was analysed by Šarkanj et al. (2018) using a stable isotope-assisted approach with enzymatic pre-treatment and solid-phase extraction. With a percentage of 19.2% positive samples and a mean of 2.37 ng DON per mL urine, a lower prevalence and lower concentrations compared with our study were observed. DH-CIT and OTA were detected in 57.5% and 78.3% of the samples with mean concentrations of 2.39 ng/mL urine and 0.05 ng/mL urine, respectively. The frequencies of occurrence of these mycotoxins are highly comparable with the herein determined occurrence rates as well as the mean concentration of OTA observed in both studies. For DH-CIT, Šarkanj et al. determined a tenfold higher concentration. In the course of the first approach to analyse TeA in urine samples by Asam et al. (2013), concentrations of 1.3 to 17.3 ng/mL in a cohort of six German volunteers were determined. Hövelmann et al. (2016) developed a method for chromatographic separation of TeA and its isomer *allo*-TeA, with mean concentrations of 6.58 ng/mL and 1.25 ng/mL in German urine samples, respectively. Urinary concentrations of TeA + *allo*-TeA in these studies are highly comparable with the values obtained with the newly developed DUS method. Nevertheless, for risk assessment, it must be considered that cytotoxic effects were intended for *allo*-TeA but not for TeA. Consequently, the potential risk for human health might be overestimated if both isomers are considered as sum parameter (Hickert et al. 2015).

The development of the first dried urine spot (DUS) method for the detection of mycotoxins provides a new and efficient tool for remote urine sampling. The sampling protocol requires minimal laboratory equipment and can easily be performed on-site in rural areas. Subsequent sample shipping is safe and eliminates the requirement for cooled transport as well as extensive safety precautions required for the shipping of liquid hazardous materials. The sample preparation protocol covering 14 mycotoxins and mycotoxin metabolites has successfully been developed and validated, showing that with the addition of stable isotope-labelled standards, a reliable analysis via HPLC-MS/MS is possible. Method validation displayed limits of detection in the range of pg/mL for most analytes, which are similar or even lower in comparison with established methods (Gerding et al. 2014), and apparent recoveries as well as repeatability were in an acceptable range for most analytes. For ZEN, as well as its metabolites ZAN, α-ZEL and β-ZEL, however, the sensitivity and ruggedness should be improved in order to be able to detect current low background exposure levels. Investigation of mycotoxin stability during storage showed that the DUS can be stored at room temperature up to 28 days without major decrease of the analyte concentration. Application of the newly developed method to the analysis of 91 Swedish urine samples allowed the detection of DH-CIT, DON, OTA and TeA in 54.9%, 75.8%, 81.2% and 98.9% of the samples, respectively. Additional analysis of the sample set with an established dilute and shoot approach indicated the increased sensitivity as well as the high accuracy of the DUS method. Taking into account the potential limitations for ZEN, ZAN, α-ZEL and β-ZEL, the new DUS method can be considered as powerful alternative sample preparation technique especially for the assessment of mycotoxin exposure in regions with reduced infrastructural opportunities. It can also be a useful complement to human biomonitoring using dried blood or serum spots as analysis of both matrices is advisable in order to evaluate short- and long-term exposure as well as the different compound-dependent metabolism.

## Supplementary Information

Below is the link to the electronic supplementary material.

Supplementary file1 (DOCX 99 KB)
