# Supplementary material for: Dried urine spots as sampling technique for multi-mycotoxin analysis in human urine
Source: Mycotoxin Res. 2021 Feb 26;37(2):129–40. doi: 10.1007/s12550-021-00423-1 (PMC8163710; doi:10.1007/s12550-021-00423-1)
Supplement: Supplementary file 1 — Supplementary file1 (DOCX 99 KB) [file 12550_2021_423_MOESM1_ESM.docx]

**Mycotoxin Research**

**Electronic Supplementary Material**

Dried urine spots as sampling technique for multi-mycotoxin analysis in human urine

Jessica Schmidt^a^, Viktoria Lindemann^a^, Monica Olsen^b3^, Benedikt Cramer^a4^, Hans-Ulrich Humpf^a*5^

^a^Institute of Food Chemistry, Westfälische Wilhelms-Universität Münster, Corrensstr. 45, 48149 Münster, Germany

^b^Risk Benefit Assessment Department, Swedish Food Agency, PO Box 622, SE-75126 Uppsala, Sweden

^*^corresponding author: [humpf@wwu.de](mailto:humpf@wwu.de)

^3^0000-0002-9049-5355

^4^0000-0001-7632-8676

^5^0000-0003-3296-3058

Table S1: sMRM parameters used for method validation and measurement of the Swedish urine samples; DP: declustering potential, CE: collision energy, CXP: collision cell exit potential, t_R_: retention time.

| Analyte | Q1 mass  [*m*/*z*] | Q3 mass [*m*/*z*] ^a^ | DP [V] | CE [eV] | CXP [V] | Expected t_R_ [min]^b^ | Mean t_R_ ± SD [min] ^c^ | Window [sec] |
| --- | --- | --- | --- | --- | --- | --- | --- | --- |
| CIT | 251 [M+H]^+^ | 91 | 75 | 56 | 13 | 7.4 | 7.60 ± 0.03 | 60 |
|  |  | 115 | 75 | 68 | 13 |  |  | 60 |
| DH-CIT | 267 [M+H]^+^ | 203 | 51 | 39 | 12 | 8.9 | 9.06 ± 0.09 | 90 |
|  |  | 231 | 51 | 32 | 12 |  |  | 90 |
| DON | 297 [M+H]^+^ | 249 | 70 | 16 | 11 | 3.2 | 3.53 ± 0.08 | 60 |
|  |  | 203 | 70 | 21 | 11 |  |  | 60 |
| FB_1_ | 722 [M+H]^+^ | 334 | 125 | 54 | 18 | 5.8 | 5.98 ± 0.02 | 30 |
|  |  | 352 | 145 | 49 | 25 |  |  | 30 |
| T-2 | 489 [M+Na]^+^ | 387 | 126 | 29 | 11 | 7.5 | 7.65 ± 0.02 | 30 |
|  |  | 245 | 126 | 37 | 11 |  |  | 30 |
| HT-2 | 442 [M+NH_4_]^+^ | 215 | 50 | 18 | 18 | 6.7 | 6.89 ± 0.02 | 30 |
|  | 447 [M+Na]^+^ | 345 | 140 | 25 | 11 |  |  | 30 |
| OTA/2'*R*-OTA | 404 [M+H]^+^ | 239 | 35 | 31 | 11 | 7.8 | 7.96/8.06 ± 0.02/0.02 | 45 |
|  |  | 221 | 35 | 48 | 11 |  |  | 45 |
| OTα | 257 [M+H]^+^ | 102 | 30 | 54 | 12 | 7.4 | 7.51 ± 0.04 | 60 |
|  |  | 193 | 20 | 37 | 23 |  |  | 60 |
| TeA + *allo*-TeA | 198 [M+H]^+^ | 125 | 50 | 23 | 11 | 6.3 | 6.51 ± 0.03 | 60 |
|  |  | 153 | 50 | 20 | 11 |  |  | 60 |
| ZAN | 319 [M-H]^-^ | 205 | -170 | -32 | -15 | 7.8 | 7.93 ± 0.03 | 30 |
|  |  | 161 | -170 | -30 | -15 |  |  | 30 |
| ZEN | 317 [M-H]^-^ | 131 | -140 | -38 | -15 | 7.8 | 7.96 ± 0.03 | 30 |
|  |  | 175 | -140 | -32 | -15 |  |  | 30 |
| α-ZEL | 319 [M-H]^-^ | 174 | -160 | -34 | -15 | 7.2 | 7.53 ± 0.02 | 90 |
|  |  | 160 | -160 | -39 | -15 |  |  | 90 |
| β-ZEL | 319 [M-H]^-^ | 174 | -160 | -34 | -15 | 7.2 | 7.18 ± 0.02 | 90 |
|  |  | 160 | -160 | -39 | -15 |  |  | 90 |
| ^13^C_3_-CIT | 254 [M+H]^+^ | 94 | 75 | 56 | 13 | 7.4 | 7.60 ± 0.03 | 60 |
|  |  | 118 | 75 | 68 | 13 |  |  | 60 |
| ^13^C_3_-DH-CIT | 270 [M+H]^+^ | 206 | 51 | 39 | 12 | 8.9 | 9.06 ± 0.09 | 90 |
|  |  | 234 | 51 | 32 | 12 |  |  | 90 |
| *d*_1_-DON | 298 [M+H]^+^ | 249 | 70 | 16 | 11 | 3.2 | 3.52± 0.08 | 60 |
|  |  | 203 | 70 | 21 | 11 |  |  | 60 |
| ^18^O_3_-FB_1_ | 728.5 [M+H]^+^ | 334 | 125 | 54 | 18 | 5.8 | 5.98 ± 0.02 | 30 |
|  |  | 352 | 145 | 49 | 25 |  |  | 30 |
| *d*_3_-T-2 | 492 [M+Na]^+^ | 390 | 126 | 29 | 11 | 7.5 | 7.64 ± 0.02 | 30 |
|  |  | 245 | 126 | 37 | 11 |  |  | 30 |
| *d*_9_-HT-2 | 451 [M+NH_4_]^+^ | 215 | 50 | 18 | 18 | 6.7 | 6.86 ± 0.02 | 30 |
|  | 456 [M+Na]^+^ | 345 | 140 | 25 | 11 |  |  | 30 |
| *d*_5_-OTA/  *d*_5_-2'*R*-OTA | 409 [M+H]^+^ | 102 | 35 | 88 | 11 |  | 7,95/8,04 ± 0,02/0,02 | 45 |
|  |  | 221 | 35 | 48 | 11 |  |  | 45 |
| ^13^C_2_-OTα | 259 [M+H]^+^ | 104 | 30 | 54 | 12 | 7.4 | 7.51 ± 0.04 | 60 |
|  | 261 [^37^Cl_1_M+H]^+^ | 141 | 20 | 46 | 18 |  |  | 60 |
|  |  |  |  |  |  |  |  |  |
| Table S1: continued from previous page. | | | | | | | | |
| Analyte | Q1 mass  [*m*/*z*] | Q3 mass [*m*/*z*] ^a^ | DP [V] | CE [eV] | CXP [V] | Exp. t_R_ [min] ^b^ | Mean t_R_ ± SD [min] ^c^ | Window [sec] |
| ^13^C_2_-TeA + *allo*-TeA | 200 [M+H]^+^ | 127 | 50 | 23 | 11 | 6.3 | 6.51 ± 0.03 | 60 |
|  |  | 155 | 50 | 20 | 11 |  |  | 60 |
| *d*_2_-ZEN | 319 [M-H]^-^ | 133 | -140 | -38 | -15 | 7.8 | 7.95 ± 0.03 | 30 |
|  |  | 177 | -140 | -32 | -15 |  |  | 30 |
| 4-Methylum-belliferyl-GlcA | 351 [M-H]^-^ | 133 | -100 | -60 | -15 | 4.8 | 4.87 ± 0.00 | 30 |
|  |  | 113 | -100 | -18 | -15 |  |  | 30 |
| 4-Methyl-umbelliferon | 177 [M+H]^+^ | 105 | 100 | 51 | 11 | 5.7 | 5.80 ± 0.01 | 30 |
|  |  | 77 | 100 | 100 | 11 |  |  | 30 |

^a^ above the quantifier is listed, below the qualifier

^b^ retention times, which were programmed for sMRM

^c^ analyte mean retention time ± standard deviation [min]

Table S2: sMRM parameters used for method development of the hydrolysis protocol; DP: declustering potential, CE: collision energy, CXP: collision cell exit potential, t_R_: retention time.

| Analyte | Q1 mass  [*m*/*z*] | Q3 mass [*m*/*z*] ^a^ | DP [V] | CE [eV] | CXP [V] | Exp. t_R_ [min] ^b^ | Mean t_R_ ± SD [min] ^c^ | Window [sec] |
| --- | --- | --- | --- | --- | --- | --- | --- | --- |
| DON-3-GlcA | 471 [M-H]^-^ | 175 | -80 | -38 | -15 | 4.0 | 3.88 ± 0.01 | 90 |
|  |  | 265 | -80 | -38 | -15 |  |  | 90 |
| HT-2-3/4-GlcA | 623 [M+Na]^+^ | 345 | 81 | 39 | 11 | 6.5 | 6.63/6.39 ± 0.00/0.00 | 60 |
|  |  | 285 | 81 | 40 | 11 |  |  | 60 |
| ZEN-GlcA | 493 [M-H]^-^ | 175 | -140 | -25 | -15 | 6.6 | 6.61 ± 0.01 | 30 |
|  |  | 317 | -140 | -30 | -9 |  |  | 30 |
| α/β-ZEL-14-GlcA | 495 [M-H]^-^ | 174 | -140 | -56 | -15 | 6.1 | 6.29/5.98 ± 0.01/0.01 | 60 |
|  |  | 160 | -140 | -65 | -15 |  |  | 60 |

^a^ above the quantifier is listed, below the qualifier

^b^ retention times, which were programmed for sMRM

^c^ analyte mean retention time ± standard deviation [min]

Table S3: Concentrations of the investigated analytes and the used stable isotope labelled standards in the calibration solutions.

| Analyte | Concentration in calibration solutions [ng/mL] | | | | | | | | | | |
| --- | --- | --- | --- | --- | --- | --- | --- | --- | --- | --- | --- |
|  | 1 | 2 | 3 | 4 | 5 | 6 | 7 | 8 | 9 | Int. Standard | 1–9 |
| CIT | 0.18 | 0.36 | 0.72 | 1.1 | 1.5 | 1.8 | 3.6 | 9.0 | 18 | ^13^C_3_-CIT | 9.0 |
| DH-CIT | 0.06 | 0.12 | 0.24 | 0.36 | 0.48 | 0.60 | 1.2 | 3.0 | 6.0 | ^13^C_3_-DH-CIT | 0.60 |
| DON | 3.0 | 6.0 | 12 | 18 | 24 | 30 | 60 | 150 | 300 | *d*_1_-DON | 18 |
| FB1 | 0.54 | 1.1 | 2.2 | 3.2 | 4.3 | 5.4 | 11 | 27 | 54 | ^18^O_3_-FB_1_ | 18 |
| T-2 | 0.30 | 0.60 | 1.2 | 1.8 | 2.4 | 3.0 | 6.0 | 15 | 30 | *d*_3_-T-2 | 3.0 |
| HT-2 | 4.5 | 9.0 | 18 | 27 | 36 | 45 | 90 | 225 | - | *d*_9_-HT-2 | 60 |
| OTA | 0.012 | 0.024 | 0.048 | 0.072 | 0.096 | 0.12 | 0.24 | 0.60 | 1.2 | *d*_5_-OTA | 0.18 |
| 2'*R*-OTA | 0.012 | 0.024 | 0.048 | 0.072 | 0.096 | 0.12 | 0.24 | 0.60 | 1.2 | *d*_5_-2'R OTA | 0.18 |
| OTα | 0.12 | 0.24 | 0.48 | 0.72 | 0.96 | 1.2 | 2.4 | 6.0 | 12 | ^13^C_2_-OTα | 1.2 |
| TeA + *allo*-TeA | 0.36 | 0.72 | 1.4 | 2.2 | 2.9 | 3.6 | 7.2 | 18 | 36 | ^13^C_2_-TeA | 6.0 |
| ZAN | 0.60 | 1.2 | 2.4 | 3.6 | 4.8 | 6.0 | 12 | 30 | 60 | *d*_2_-ZEN | 6.0 |
| ZEN | 0.30 | 0.60 | 1.2 | 1.8 | 2.4 | 3.0 | 6.0 | 15 | 30 | *d*_2_-ZEN | 6.0 |
| α-ZEL | 0.60 | 1.2 | 2.4 | 3.6 | 4.8 | 6.0 | 12 | 30 | 60 | *d*_2_-ZEN | 6.0 |
| β-ZEL | 0.84 | 1.7 | 3.4 | 5.0 | 6.7 | 8.4 | 17 | 42 | 84 | *d*_2_-ZEN | 6.0 |

Table S4: Mean relative analyte recovery rate ± standard deviation [%] after blank urine was spiked at medium concentration levels and stored as dried urine spots for 7, 14 and 28 days at 22 , 4  and -18 °C; *n*=4 for each storage time and temperature.

| Storage time | c  [ng/mL urine] | 7 days | | | 14 days | | | 28 days | | |
| --- | --- | --- | --- | --- | --- | --- | --- | --- | --- | --- |
| Temperature |  | 22 °C | 4 °C | -18 °C | 22 °C | 4 °C | -18 °C | 22 °C | 4 °C | -18 °C |
| Analyte |  |  |  |  |  |  |  |  |  |  |
| CIT | 1.8 | 82.9 ± 6.6 | 92.3 ± 4.1 | 94.7 ± 4.9 | 84.3 ± 4.3 | 83.8 ± 7.6 | 86.8 ± 4.1 | 77.6 ±6.5 | 86.5 ± 5.0 | 88.2 ± 2.9 |
| DH-CIT | 0.6 | 101.1 ± 4.5 | 107.7 ± 3.8 | 111.0 ± 4.5 | 107.3 ± 2.9 | 103.8 ± 5.9 | 100.3 ± 3.5 | 101.3 ± 8.9 | 109.0 ± 4.0 | 105.7 ± 4.8 |
| DON | 30 | 99.5 ± 2.6 | 103.2 ± 2.3 | 102.9 ± 2.0 | 104.3 ± 3.8 | 98.4 ± 7.0 | 95.4 ± 6.6 | 99.1 ± 6.2 | 102.9 ± 2.6 | 100.3 ± 1.9 |
| FB_1_ | 5.4 | 36.7 ± 3.5 | 34.0 ± 3.4 | 44.1 ± 7.2 | 51.6 ± 4.8 | 49.7 ± 6.6 | 51.5 ± 9.5 | 113.6 ± 6.8 | 116.4 ± 5.2 | 108.6 ± 1.2 |
| T-2 | 3 | 89.8 ± 4.7 | 98.4 ± 3.5 | 100.6 ± 9.8 | 94.0 ± 3.1 | 90.0 ± 10.3 | 90.8 ± 8.4 | 99.1 ± 13.1 | 100.6 ± 7.1 | 97.6 ± 5.5 |
| HT-2 | 45 | 98.7 ± 6.6 | 110.1 ± 7.8 | 113.7 ± 11.5 | 106.5 ± 12.5 | 95.9 ± 8.5 | 90.0 ± 11.1 | 96.5 ± 9.6 | 103.6 ± 9.8 | 101.8 ± 12.3 |
| OTA | 0.12 | 96.5 ± 3.9 | 104.1 ± 12.6 | 108.2 ± 9.8 | 103.1 ± 14.3 | 86.1 ± 10.0 | 96.0 ± 7.1 | 110.0 ± 16.1 | 107.7 ± 11.7 | 101.7 ± 3.5 |
| 2’*R*-OTA | 0.12 | 86.7 ± 6.1 | 99.7 ± 9.0 | 101.2 ± 6.8 | 102.3 ± 10.5 | 92.8 ± 11.6 | 89.8 ± 5.9 | 106.7 ± 11.8 | 101.1 ± 4.1 | 96.9 ± 0.5 |
| OTα | 1.2 | 95.3 ± 5.6 | 101.7 ± 3.3 | 106.4 ± 4.7 | 100.8 ± 1.4 | 96.8 ± 11.3 | 97.6 ± 3.1 | 94.8 ± 7.1 | 102.6 ± 5.8 | 100.0 ± 5.3 |
| TeA + *allo*-TeA | 3.6 | 92.7 ± 2.9 | 94.2 ± 2.7 | 94.7 ± 4.7 | 99.7 ± 5.0 | 95.4 ± 2.7 | 93.8 ± 3.5 | 92.4 ± 2.0 | 92.7 ± 3.7 | 93.3 ± 4.3 |
| ZAN | 6 | 68.3 ± 13.4 | 82.5 ± 10.3 | 83.9 ± 10.4 | 77.9 ± 17.0 | 72.0 ± 31.7 | 83.8 ± 9.2 | 79.7 ± 12.3 | 88.2 ± 15.7 | 73.8 ± 20.6 |
| ZEN | 3 | 61.3 ± 25.3 | 72.7 ± 17.6 | 58.9 ± 21.8 | 66.0 ± 15.7 | 74.8 ± 12.7 | 88.8 ± 39.2 | 70.1 ± 10.9 | 72.0 ± 19.8 | 56.6 ± 21.5 |
| α-ZEL | 6 | 61.8 ± 17.2 | 85.0 ± 10.8 | 82.9 ± 22.2 | 88.2 ± 36.2 | 88.7 ± 21.8 | 81.9 ± 23.4 | 92.5 ± 40.4 | 110.3 ± 33.8 | 69.9 ± 14.1 |
| β-ZEL | 8.4 | 25.1 ± 11.3 | 36.9 ± 21.3 | 32.2 ± 21.3 | 39.6 ± 6.6 | 55.2 ± 21.1 | 58.4 ± 24.7 | 41.5 ± 12.6 | 58.8 ± 14.3 | 62.3 ± 29.6 |

Table S5: Mycotoxin concentration in Swedish urine samples determined by application of the newly developed DUS method adjusted for creatinine (*n*=91).

| Mycotoxin | Positive samples % (n) | Quantitated samples % (n) ^d^ | Concentration [ng/mg creatinine] | | | |
| --- | --- | --- | --- | --- | --- | --- |
|  |  |  | Mean ^a^ | Median ^a^ | Maximum | SD ^b^ quanti-tated samples |
| DH-CIT | 54.9 (50) | 38.5 (35) | 0.18 | 0.10 | 0.70 | 0.16 |
| DON | 75.8 (69) | 70.3 (64) | 16 | 10 | 85 | 16 |
| OTA | 80.2 (73) | 67.0 (61) | 0.040 | 0.030 | 0.230 | 0.030 |
| TeA+ *allo*-TeA | 97.8 (89) | 93.4 (85) | 4.3 | 2.4 | 30 ^c^ | 5.4 |

^a^ Mean and median were calculated from samples with mycotoxin concentrations > LOQ

^b^ SD: standard deviation

^c^ value was calculated by extrapolation of the calibration curve

^d^ for one sample creatinine was not determined

Table S6: Detailed list of the determined mycotoxin concentrations in 91 Swedish urine samples obtained with the DUS approach. Each sample was analysed in duplicate.

| Sample number | Mean concentration (range) | | | | | | | |
| --- | --- | --- | --- | --- | --- | --- | --- | --- |
|  | DH-CIT | | DON | | OTA | | TeA + *allo-*TeA | |
|  | [ng/mL urine] | [ng/mg creatinine] | [ng/mL urine] | [ng/mg creatinine] | [ng/mL urine] | [ng/mg creatinine] | [ng/mL urine] | [ng/mg creatinine] |
| 1 | 0.07 (0.01) | 0.08 (0.01) | <LOQ | < LOQ | 0.037 (0.003) | 0.044 (0.004) | 1.9 (<0.1) | 2.2 (<0.1) |
| 2 | <LOQ | < LOQ | 3.1 (0.1) | 4.4 (0.1) | 0.038 (0.002) | 0.053 (0.003) | 5.0 (<0.1) | 7.1 (<0.1) |
| 3 | 0.10 (<0.01) | 0.06 (<0.01) | 6.8 (<0.1) | 4.2 (<0.1) | 0.060 (<0.001) | 0.037 (<0.001) | 5.2 (0.1) | 3.2 (0.1) |
| 4 | <LOQ | < LOQ | n.d. | n.d. | 0.063 (0.006) | 0.046 (0.004) | 3.7 (0.2) | 2.7 (0.1) |
| 5 | 0.26 (<0.01) | 0.15 (<0.01) | n.d. | n.d. | 0.049 (0.003) | 0.028 (0.001) | 9.9 (0.1) | 5.6 (<0.1) |
| 6 | 0.08 (<0.01) | 0.06 (<0.01) | 7.3 (0.2) | 5.5 (0.1) | 0.041 (0.005) | 0.03 (0.003) | 0.42 (0.03) | 0.31 (0.02) |
| 7 | n.d. | n.d. | 20 (1) | 8.6 (0.6) | 0.078 (0.001) | 0.034 (0.001) | 0.86 (0.02) | 0.38 (0.01) |
| 8 | 0.07 (<0.01) | 0.03 (<0.01) | 13 (<1) | 5.1 (0.2) | 0.127 (0.001) | 0.05 (<0.001) | 0.75 (0.05) | 0.30 (0.02) |
| 9 | <LOQ | < LOQ | 6.8 (<0.1) | 5.5 (<0.1) | 0.050 (0.004) | 0.04 (0.003) | 1.2 (0.1) | 0.95 (0.05) |
| 10 | <LOQ | < LOQ | 8.6 (0.2) | 3.8 (<0.1) | 0.051 (0.001) | 0.023 (0.001) | <LOQ | < LOQ |
| 11 | n.d. | n.d. | 5.2 (0.2) | 3.3 (0.1) | 0.035 (0.004) | 0.022 (0.003) | <LOQ | < LOQ |
| 12 | <LOQ | < LOQ | 3.4 (0.1) | 3.9 (0.1) | 0.033 (<0.001) | 0.038 (<0.001) | 2.1 (0.1) | 2.5 (0.1) |
| 13 | n.d. | n.d. | <LOQ | < LOQ | 0.037 (0.002) | 0.042 (0.003) | 0.38 (0.02) | 0.43 (0.02) |
| 14 | 0.41 (0.03) | 0.17 (0.01) | 63 (3) | 26 (1) | 0.124 (0.006) | 0.052 (0.003) | 6.2 (0.3) | 2.6 (0.1) |
| 15 | n.d. | n.d. | n.d. | n.d. | 0.063 (0.002) | 0.056 (0.001) | n.d. | n.d. |
| 16 | <LOQ | < LOQ | n.d. | n.d. | 0.046 (<0.001) | 0.034 (<0.001) | 3.1 (0.3) | 2.3 (0.2) |
| 17 | <LOQ | < LOQ | n.d. | n.d. | 0.036 (0.002) | 0.029 (0.002) | 0.56 (0.02) | 0.44 (0.02) |
| 18 | <LOQ | < LOQ | <LOQ | < LOQ | 0.041 (0.004) | 0.039 (0.004) | 13 (1) | 12 (1) |
| 19 | <LOQ | < LOQ | <LOQ | < LOQ | 0.036 (0.001) | 0.040 (0.001) | 6.9 (0.3) | 7.7 (0.4) |
| 20 | n.d. | n.d. | 3.8 (0.5) | 5.9 (0.8) | 0.027 (0.001) | 0.041 (0.002) | 5.7 (<0.1) | 8.6 (<0.1) |
| 21 | 0.10 (0.01) | 0.16 (0.01) | n.d. | n.d. | 0.032 (0.002) | 0.052 (0.004) | 4.1 (<0.1) | 6.7 (0.1) |
| 22 | n.d. | n.d. | 4.4 (0.1) | 2.7 (<0.1) | 0.022 (0.002) | 0.014 (0.001) | 1.2 (<0.1) | 0.76 (0.01) |
| 23 | <LOQ | < LOQ | 17 (<1) | 8.1 (0.3) | <LOQ | < LOQ | 2.4 (0.1) | 1.2 (0.1) |
| 24 | n.d. | n.d. | 4.3 (0.4) | 4.3 (0.4) | 0.021 (0.003) | 0.021 (0.003) | 1.3 (<0.1) | 1.2 (<0.1) |
| 25 | n.d. | n.d. | 5.8 (0.3) | 2.7 (0.2) | 0.031 (0.003) | 0.015 (0.001) | 9.0 (0.2) | 4.2 (0.1) |
| 26 | 0.26 (0.02) | 0.04 (<0.01) | n.d. | n.d. | 0.103 (0.015) | 0.015 (0.002) | 0.58 (0.02) | 0.08 (<0.01) |
| Table S6: continued from previous page. | | | | | | | | |
| Sample number | Mean concentration (range) | | | | | | | |
|  | DH-CIT | | DON | | OTA | | TeA + *allo-*TeA | |
|  | [ng/mL urine] | [ng/mg creatinine] | [ng/mL urine] | [ng/mg creatinine] | [ng/mL urine] | [ng/mg creatinine] | [ng/mL urine] | [ng/mg creatinine] |
| 27 | n.d. | n.d. | n.d. | n.d. | 0.027 (0.002) | 0.012 (0.001) | 1.2 (<0.1) | 0.51 (<0.01) |
| 28 | 0.10 (0.01) | 0.03 (<0.01) | n.d. | n.d. | 0.051 (0.006) | 0.015 (0.002) | 1.3 (0.1) | 0.38 (0.03) |
| 29 | 0.14 (0.01) | 0.05 (<0.01) | 6.1 (0.2) | 2.0 (0.1) | 0.040 (0.002) | 0.013 (0.001) | 5.4 (0.5) | 1.8 (0.2) |
| 30 | 0.74 (0.01) | 0.70 (0.01) | 90 (3) | 85 (3) | 0.023 (0.001) | 0.021 (0.001) | 0.89 (<0.01) | 0.84 (<0.01) |
| 31 | 0.10 (<0.01) | 0.08 (<0.01) | n.d. | n.d. | 0.020 (0.003) | 0.016 (0.002) | 1.3 (<0.1) | 1.0 (<0.1) |
| 32 | 0.13 (<0.01) | 0.06 (<0.01) | 11 (<1) | 4.7 (0.1) | 0.092 (0.005) | 0.039 (0.002) | 1.9 (0.1) | 0.81 (0.05) |
| 33 | n.d. | n.d. | n.d. | n.d. | 0.086 (0.007) | 0.036 (0.003) | 0.73 (<0.01) | 0.31 (<0.01) |
| 34 | n.d. | n.d. | 7.7 (0.1) | 7.9 (0.1) | n.d. | n.d. | 15 (<1) | 15 (<1) |
| 35 | 0.07 (0.01) | 0.08 (0.01) | 13 (<1) | 15 (<1) | 0.025 (<0.01) | 0.028 (<0.001) | 8.2 (0.1) | 9.2 (0.1) |
| 36 | n.d. | n.d. | 11 (<1) | 11 (<1) | 0.033 (0.001) | 0.035 (0.001) | 1.1 (<0.1) | 1.2 (<0.1) |
| 37 | n.d. | n.d. | 12 (<1) | 13 (<1) | <LOQ | < LOQ | 2.5 (<0.1) | 2.6 (<0.1) |
| 38 | <LOQ | < LOQ | 112 (<1) | 9.8 (0.2) | 0.023 (0.002) | 0.019 (0.002) | 3.7 (<0.1) | 3.2 (<0.1) |
| 39 | n.d. | n.d. | 44 (1) | 31 (1) | 0.021 (<0.01) | 0.015 (<0.001) | 1.1 (<0.1) | 0.75 (0.01) |
| 40 | n.d. | n.d. | 9.5 (0.2) | 18 (<1) | n.d. | n.d. | 0.76 (<0.01) | 1.4 (<0.1) |
| 41 | n.d. | n.d. | 33 (1) | 23 (1) | 0.038 (0.003) | 0.028 (0.002) | 1.4 (<0.1) | 0.98 (<0.01) |
| 42 | n.d. | n.d. | 18 (<1) | 22 (1) | 0.020 (0.001) | 0.025 (0.001) | 1.2 (0.1) | 1.5 (0.1) |
| 43 | n.d. | n.d. | 8.8 (0.2) | 9.8 (0.3) | <LOQ | < LOQ | 1.0 (<0.1) | 1.1 (<0.1) |
| 44 | n.d. | n.d. | 17 (1) | 14 (1) | <LOQ | < LOQ | 6.4 (0.2) | 5.3 (0.2) |
| 45 | <LOQ | ^a^ | n.d. | ^a^ | 0.020 (0.002) | ^a^ | 4.0 (0.1) | ^a^ |
| 46 | 0.52 (0.04) | 0.19 (0.01) | n.d. | n.d. | 0.023 (0.004) | 0.008 (0.001) | 11 (<1) | 3.9 (0.2) |
| 47 | n.d. | n.d. | 8.4 (0.3) | 6.2 (0.2) | 0.019 (<0.001) | 0.014 (<0.001) | 3.9 (0.1) | 2.8 (<0.1) |
| 48 | 0.27 (<0.01) | 0.29 (<0.01) | 24 (1) | 25 (1) | n.d. | n.d. | 6.5 (0.6) | 6.9 (0.6) |
| 49 | n.d. | n.d. | 8.8 (0.4) | 6.2 (0.3) | 0.022 (0.001) | 0.016 (0.001) | 8.1 (0.3) | 5.7 (0.2) |
| 50 | <LOQ | <LOQ | 19 (<1) | 25 (<1) | n.d. | n.d. | 23 (1) | 30 (1) |
| 51 | n.d. | n.d. | 4.3 (0.2) | 4.7 (0.2) | 0.025 (0.005) | 0.027 (0.006) | 3.9 (0.2) | 4.2 (0.2) |
| 52 | <LOQ | <LOQ | 21 (1) | 19 (<1) | n.d. | n.d. | 2.7 (<0.1) | 2.4 (<0.1) |
| 53 | n.d. | n.d. | 5.8 (0.9) | 3.1 (0.5) | 0.434 (0.027) | 0.232 (0.015) | 1.7 (<0.1) | 0.92 (0.02) |
| Table S6: continued from previous page. | | | | | | | | |
| Sample number | Mean concentration (range) | | | | | | | |
|  | DH-CIT | | DON | | OTA | | TeA + *allo-*TeA | |
|  | [ng/mL urine] | [ng/mg creatinine] | [ng/mL urine] | [ng/mg creatinine] | [ng/mL urine] | [ng/mg creatinine] | [ng/mL urine] | [ng/mg creatinine] |
| 54 | 0.63 (0.03) | 0.34 (0.02) | n.d. | n.d. | n.d. | n.d. | 4.0 (0.1) | 2.1 (<0.1) |
| 55 | n.d. | n.d. | 19 (<1) | 13 (<1) | n.d. | n.d. | 5.5 (<0.1) | 3.6 (<0.1) |
| 56 | n.d. | n.d. | 57 (2) | 21 (1) | <LOQ | < LOQ | 14 (<1) | 5.2 (0.1) |
| 57 | n.d. | n.d. | 22 (1) | 53 (1) | n.d. | n.d. | 5.1 (0.1) | 12 (<1) |
| 58 | 0.77 (0.04) | 0.31 (0.01) | 12 (<1) | 4.8 (<0.1) | 0.028 (0.001) | 0.011 (<0.001) | 15 (<1) | 5.8 (0.1) |
| 59 | n.d. | n.d. | 6.4 (3.7) | 13 (8) | n.d. | n.d. | 1.6 (0.1) | 3.2 (0.2) |
| 60 | <LOQ | <LOQ | 12 (1) | 24 (1) | n.d. | n.d. | 0.78 (0.01) | 1.6 (<0.1) |
| 61 | <LOQ | <LOQ | 11 (1) | 11 (1) | n.d. | n.d. | 1.1 (<0.1) | 1.1 (<0.1) |
| 62 | n.d. | n.d. | 11 (1) | 6.3 (0.3) | <LOQ | < LOQ | 37 (1) ^b^ | 22 (<1) ^b^ |
| 63 | 0.10 (<0.01) | 0.09 (<0.01) | 12 (<1) | 10 (<1) | n.d. | n.d. | 6.6 (0.1) | 5.8 (0.1) |
| 64 | 0.55 (0.01) | 0.36 (0.01) | n.d. | n.d. | 0.039 (0.009) | 0.025 (0.006) | 1.4 (<0.1) | 0.93 (0.03) |
| 65 | n.d. | n.d. | n.d. | n.d. | 0.127 (0.023) | 0.056 (0.010) | 43 (<1) ^b^ | 19 (<1) ^b^ |
| 66 | 0.18 (<0.01) | 0.05 (<0.01) | 136 (2) | 42 (1) | 0.101 (<0.001) | 0.031 (<0.001) | 1.3 (<0.1) | 0.40 (0.01) |
| 67 | n.d. | n.d. | 3.8 (0.6) | 3.1 (0.5) | <LOQ | < LOQ | 1.1 (0.1) | 0.87 (0.04) |
| 68 | 0.07 (<0.01) | 0.07 (<0.01) | 11 (<1) | 10 (<1) | n.d. | n.d. | 0.64 (0.03) | 0.62 (0.03) |
| 69 | 0.09 (0.01) | 0.06 (<0.01) | 124 (4) | 77 (2) | 0.059 (0.007) | 0.037 (0.004) | 3.9 (0.1) | 2.4 (0.1) |
| 70 | 0.17 (0.02) | 0.24 (0.02) | 9.9 (0.1) | 14 (<1) | n.d. | n.d. | 2.2 (0.1) | 3.0 (0.2) |
| 71 | n.d. | n.d. | 15 (1) | 9.6 (0.7) | 0.077 (0.013) | 0.050 (0.008) | 0.73 (0.04) | 0.47 (0.02) |
| 72 | n.d. | n.d. | 49 (2) | 24 (1) | 0.053 (0.001) | 0.026 (<0.001) | 26 (1) | 13 (<1) |
| 73 | n.d. | n.d. | 31 (2) | 20 (1) | n.d. | n.d. | 6.3 (0.6) | 4.0 (0.4) |
| 74 | n.d. | n.d. | 25 (1) | 30 (1) | n.d. | n.d. | 2.3 (<0.1) | 2.7 (<0.1) |
| 75 | 0.06 (<0.01) | 0.06 (<0.01) | 36 (<1) | 35 (<1) | <LOQ | < LOQ | 2.1 (<0.1) | 2.0 (<0.1) |
| 76 | n.d. | n.d. | 17 (<1) | 41 (<1) | <LOQ | < LOQ | 3.6 (<0.1) | 8.6 (<0.1) |
| 77 | 0.43 (0.03) | 0.16 (0.01) | n.d. | n.d. | 0.050 (0.003) | 0.019 (0.001) | 10 (<1) | 3.9 (0.2) |
| 78 | 0.52 (0.01) | 0.19 (0.01) | n.d. | n.d. | 0.051 (0.006) | 0.018 (0.002) | <LOQ | < LOQ |
| 79 | n.d. | n.d. | n.d. | n.d. | 0.031 (0.001) | 0.036 (0.001) | <LOQ | < LOQ |
| 80 | n.d. | n.d. | 8.3 (0.3) | 8.5 (0.3) | 0.022 (0.004) | 0.022 (0.004) | 0.90 (0.04) | 0.93 (0.04) |
| Table S6: continued from previous page. | | | | | | | | |
| Sample number | Mean concentration (range) | | | | | | | |
|  | DH-CIT | | DON | | OTA | | TeA + *allo-*TeA | |
|  | [ng/mL urine] | [ng/mg creatinine] | [ng/mL urine] | [ng/mg creatinine] | [ng/mL urine] | [ng/mg creatinine] | [ng/mL urine] | [ng/mg creatinine] |
| 81 | 0.60 (0.01) | 0.27 (0.01) | 13 (<1) | 6.0 (0.1) | 0.088 (0.001) | 0.040 (<0.001) | 3.9 (0.3) | 1.8 (0.2) |
| 82 | 0.39 (0.01) | 0.37 (0.01) | n.d. | n.d. | n.d. | n.d. | 7.8 (0.2) | 7.5 (0.2) |
| 83 | 0.10 (<0.01) | 0.05 (<0.01) | 37 (<1) | 18 (<1) | 0.062 (0.001) | 0.030 (<0.001) | 47 (<1) ^b^ | 22 (<1) ^b^ |
| 84 | n.d. | n.d. | 54 (4) | 46 (3) | 0.047 (0.014) | 0.040 (0.012) | 6.4 (0.8) | 5.5 (0.7) |
| 85 | n.d. | n.d. | 9.6 (0.2) | 16 (<1) | 0.288 (0.003) | 0.142 (0.002) | 1.9 (<0.1) | 0.94 (0.02) |
| 86 | 0.97 (0.02) | 0.44 (0.01) | n.d. | n.d. | 0.053 (0.005) | 0.024 (0.002) | 5.3 (0.1) | 2.4 (0.1) |
| 87 | 0.44 (<0.01) | 0.61 (<0.01) | n.d. | n.d. | <LOQ | < LOQ | 0.65 (0.01) | 0.90 (0.02) |
| 88 | n.d. | n.d. | 9.6 (0.2) | 5.7 (0.1) | <LOQ | < LOQ | 16 (<1) | 9.3 (0.1) |
| 89 | 0.07 (0.01) | 0.06 (<0.01) | 23 (<1) | 18 (<1) | 0.040 (0.002) | 0.032 (0.002) | 6.7 (<0.1) | 5.3 (0.1) |
| 90 | n.d. | n.d. | <LOQ | < LOQ | <LOQ | < LOQ | 3.5 (0.1) | 4.2 (0.1) |
| 91 | 0.23 (0.02) | 0.10 (0.01) | 14 (1) | 6.2 (0.5) | 0.078 (0.003) | 0.035 (0.001) | 4.2 (0.1) | 1.9 (<0.1) |

^a^ creatinine value not determined

^b^ values were calculated by extrapolation of the calibration curve

Table S7: Detailed list of the determined mycotoxin concentrations in 91 Swedish urine samples obtained with the DaS approach (Warensjö Lemming et al. 2020). Each sample was analysed in duplicate.

| Sample number | Mean concentration (range) | | | | | | | |
| --- | --- | --- | --- | --- | --- | --- | --- | --- |
|  | DH-CIT | | DON | | DON-GlcA | | OTA | |
|  | [ng/mL urine] | [ng/mg creatinine] | [ng/mL urine] | [ng/mg creatinine] | [ng/mL urine] | [ng/mg creatinine] | [ng/mL urine] | [ng/mg creatinine] |
| 1 | n.d. | n.d. | n.d. | n.d. | n.d. | n.d. | n.d. | n.d. |
| 2 | n.d. | n.d. | n.d. | n.d. | n.d. | n.d. | n.d. | n.d. |
| 3 | n.d. | n.d. | n.d. | n.d. | n.d. | n.d. | n.d. | n.d. |
| 4 | n.d. | n.d. | n.d. | n.d. | n.d. | n.d. | n.d. | n.d. |
| 5 | n.d. | n.d. | n.d. | n.d. | n.d. | n.d. | n.d. | n.d. |
| 6 | n.d. | n.d. | n.d. | n.d. | n.d. | n.d. | n.d. | n.d. |
| 7 | n.d. | n.d. | n.d. | n.d. | n.d. | n.d. | n.d. | n.d. |
| 8 | n.d. | n.d. | n.d. | n.d. | n.d. | n.d. | n.d. | n.d. |
| 9 | n.d. | n.d. | n.d. | n.d. | n.d. | n.d. | n.d. | n.d. |
| 10 | n.d. | n.d. | n.d. | n.d. | n.d. | n.d. | n.d. | n.d. |
| 11 | n.d. | n.d. | n.d. | n.d. | n.d. | n.d. | n.d. | n.d. |
| 12 | n.d. | n.d. | n.d. | n.d. | n.d. | n.d. | n.d. | n.d. |
| 13 | n.d. | n.d. | n.d. | n.d. | n.d. | n.d. | n.d. | n.d. |
| 14 | <LOQ | < LOQ | n.d. | n.d. | n.d. | n.d. | n.d. | n.d. |
| 15 | n.d. | n.d. | n.d. | n.d. | n.d. | n.d. | n.d. | n.d. |
| 16 | n.d. | n.d. | n.d. | n.d. | n.d. | n.d. | n.d. | n.d. |
| 17 | n.d. | n.d. | n.d. | n.d. | n.d. | n.d. | n.d. | n.d. |
| 18 | n.d. | n.d. | n.d. | n.d. | n.d. | n.d. | n.d. | n.d. |
| 19 | n.d. | n.d. | n.d. | n.d. | n.d. | n.d. | n.d. | n.d. |
| 20 | n.d. | n.d. | n.d. | n.d. | 9.0 (1.3) | 13 (2) | n.d. | n.d. |
| 21 | n.d. | n.d. | n.d. | n.d. | n.d. | n.d. | n.d. | n.d. |
| 22 | n.d. | n.d. | n.d. | n.d. | n.d. | n.d. | n.d. | n.d. |
| 23 | n.d. | n.d. | 17 (1) | 8.4 (0.5) | 22 (1) | 11 (<1) | n.d. | n.d. |
| 24 | n.d. | n.d. | n.d. | n.d. | n.d. | n.d. | n.d. | n.d. |
| 25 | n.d. | n.d. | n.d. | n.d. | n.d. | n.d. | n.d. | n.d. |
| 26 | n.d. | n.d. | n.d. | n.d. | n.d. | n.d. | n.d. | n.d. |
| Table S7: continued from previous page. | | | | | | | | |
| Sample number | Mean concentration (range) | | | | | | | |
|  | DH-CIT | | DON | | DON-GlcA | | OTA | |
|  | [ng/mL urine] | [ng/mg creatinine] | [ng/mL urine] | [ng/mg creatinine] | [ng/mL urine] | [ng/mg creatinine] | [ng/mL urine] | [ng/mg creatinine] |
| 27 | n.d. | n.d. | n.d. | n.d. | n.d. | n.d. | n.d. | n.d. |
| 28 | n.d. | n.d. | n.d. | n.d. | n.d. | n.d. | n.d. | n.d. |
| 29 | n.d. | n.d. | n.d. | n.d. | n.d. | n.d. | n.d. | n.d. |
| 30 | <LOQ | < LOQ | 82 (3) | 77 (3) | 45 (1) | 43 (1) | n.d. | n.d. |
| 31 | n.d. | n.d. | n.d. | n.d. | n.d. | n.d. | n.d. | n.d. |
| 32 | n.d. | n.d. | n.d. | n.d. | n.d. | n.d. | n.d. | n.d. |
| 33 | n.d. | n.d. | n.d. | n.d. | n.d. | n.d. | n.d. | n.d. |
| 34 | n.d. | n.d. | n.d. | n.d. | n.d. | n.d. | n.d. | n.d. |
| 35 | n.d. | n.d. | n.d. | n.d. | 10 (<1) | 12 (<1) | n.d. | n.d. |
| 36 | n.d. | n.d. | <LOQ | < LOQ | 12 (1) | 12 (1) | n.d. | n.d. |
| 37 | n.d. | n.d. | n.d. | n.d. | 7.8 (0.4) | 8.1 (0.4) | n.d. | n.d. |
| 38 | n.d. | n.d. | n.d. | n.d. | 12 (1) | 10 (1) | n.d. | n.d. |
| 39 | n.d. | n.d. | 17 (7) | 12 (5) | 23 (1) | 16 (1) | n.d. | n.d. |
| 40 | n.d. | n.d. | n.d. | n.d. | 11 (<1) | 21 (1) | n.d. | n.d. |
| 41 | n.d. | n.d. | <LOQ | < LOQ | n.d. | n.d. | n.d. | n.d. |
| 42 | n.d. | n.d. | n.d. | n.d. | 15 (2) | 19 (2) | n.d. | n.d. |
| 43 | n.d. | n.d. | n.d. | n.d. | 5.0 (1.1) | 5.7 (1.3) | n.d. | n.d. |
| 44 | n.d. | n.d. | n.d. | n.d. | 25 (1) | 21 (<1) | n.d. | n.d. |
| 45 | n.d. | ^a^ | n.d. | ^a^ | 4.7 (0.5) | ^a^ | n.d. | ^a^ |
| 46 | <LOQ | < LOQ | <LOQ | < LOQ | n.d. | n.d. | n.d. | n.d. |
| 47 | n.d. | n.d. | <LOQ | < LOQ | n.d. | n.d. | n.d. | n.d. |
| 48 | n.d. | n.d. | 32 (<1) | 35 (<1) | 32 (1) | 34 (1) | n.d. | n.d. |
| 49 | n.d. | n.d. | <LOQ | < LOQ | n.d. | n.d. | n.d. | n.d. |
| 50 | n.d. | n.d. | <LOQ | < LOQ | 24 (1) | 31 (1) | n.d. | n.d. |
| 51 | n.d. | n.d. | <LOQ | < LOQ | n.d. | n.d. | n.d. | n.d. |
| 52 | n.d. | n.d. | <LOQ | < LOQ | 25 (4) | 22 (4) | n.d. | n.d. |
| 53 | n.d. | n.d. | n.d. | n.d. | n.d. | n.d. | <LOQ | <LOQ |
| Table S7: continued from previous page. | | | | | | | | |
| Sample number | Mean concentration (range) | | | | | | | |
|  | DH-CIT | | DON | | DON-GlcA | | OTA | |
|  | [ng/mL urine] | [ng/mg creatinine] | [ng/mL urine] | [ng/mg creatinine] | [ng/mL urine] | [ng/mg creatinine] | [ng/mL urine] | [ng/mg creatinine] |
| 54 | <LOQ | < LOQ | n.d. | n.d. | 14 (4) | 7.4 (2.5) | n.d. | n.d. |
| 55 | n.d. | n.d. | <LOQ | < LOQ | 11 (20) | 7.0 (1.2) | n.d. | n.d. |
| 56 | n.d. | n.d. | <LOQ | < LOQ | 22 (1) | 8.0 (3.4) | n.d. | n.d. |
| 57 | n.d. | n.d. | 19 (1) | 73 (3) | 30 (1) | 73 (3) | n.d. | n.d. |
| 58 | <LOQ | < LOQ | n.d. | n.d. | n.d. | n.d. | n.d. | n.d. |
| 59 | n.d. | n.d. | <LOQ | < LOQ | 5.0 (1.2) | 13 (25) | n.d. | n.d. |
| 60 | n.d. | n.d. | 18 (1) | 37 (2) | n.d. | n.d. | n.d. | n.d. |
| 61 | n.d. | n.d. | n.d. | n.d. | 9.3 (0.6) | 9.0 (0.5) | n.d. | n.d. |
| 62 | n.d. | n.d. | <LOQ | < LOQ | 22 (1) | 13 (<1) | n.d. | n.d. |
| 63 | n.d. | n.d. | n.d. | n.d. | 14 (1) | 13 (1) | n.d. | n.d. |
| 64 | <LOQ | < LOQ | n.d. | n.d. | n.d. | n.d. | n.d. | n.d. |
| 65 | <LOQ | < LOQ | n.d. | n.d. | n.d. | n.d. | n.d. | n.d. |
| 66 | n.d. | n.d. | 47 (2) | 14 (1) | 60 (3) | 19 (1) | n.d. | n.d. |
| 67 | n.d. | n.d. | n.d. | < LOQ | 8.6 (1.6) | 7.1 (1.4) | n.d. | n.d. |
| 68 | n.d. | n.d. | n.d. | < LOQ | 12 (<1) | 11 (<1) | n.d. | n.d. |
| 69 | n.d. | n.d. | 102 (2) | 64 (1) | 58 (10) | 36 (6) | n.d. | n.d. |
| 70 | n.d. | n.d. | n.d. | < LOQ | 15 (<1) | 21 (<1) | n.d. | n.d. |
| 71 | n.d. | n.d. | n.d. | < LOQ | n.d. | n.d. | n.d. | n.d. |
| 72 | n.d. | n.d. | 34 (3) | 16 (2) | 29 (3) | 14 (1) | n.d. | n.d. |
| 73 | n.d. | n.d. | 22 (3) | 14 (2) | 31 (1) | 19 (1) | n.d. | n.d. |
| 74 | n.d. | n.d. | <LOQ | < LOQ | n.d. | n.d. | n.d. | n.d. |
| 75 | n.d. | n.d. | 28 (1) | 27 (1) | 20 (4) | 19 (6) | n.d. | n.d. |
| 76 | n.d. | n.d. | 21 (2) | 50 (6) | 19 (1) | 44 (3) | n.d. | n.d. |
| 77 | <LOQ | < LOQ | n.d. | n.d. | n.d. | n.d. | n.d. | n.d. |
| 78 | <LOQ | < LOQ | n.d. | n.d. | n.d. | n.d. | n.d. | n.d. |
| 79 | n.d. | n.d. | n.d. | n.d. | <LOQ | < LOQ | n.d. | n.d. |
| 80 | n.d. | n.d. | <LOQ | < LOQ | n.d. | n.d. | n.d. | n.d. |
| Table S7: continued from previous page. | | | | | | | | |
| Sample number | Mean concentration (range) | | | | | | | |
|  | DH-CIT | | DON | | DON-GlcA | | OTA | |
|  | [ng/mL urine] | [ng/mg creatinine] | [ng/mL urine] | [ng/mg creatinine] | [ng/mL urine] | [ng/mg creatinine] | [ng/mL urine] | [ng/mg creatinine] |
| 81 | <LOQ | < LOQ | n.d. | n.d. | n.d. | n.d. | n.d. | n.d. |
| 82 | <LOQ | < LOQ | n.d. | n.d. | n.d. | n.d. | n.d. | n.d. |
| 83 | <LOQ | < LOQ | n.d. | n.d. | 16 (1) | 7.6 (0.6) | n.d. | n.d. |
| 84 | n.d. | n.d. | <LOQ | < LOQ | n.d. | n.d. | n.d. | n.d. |
| 85 | n.d. | n.d. | <LOQ | < LOQ | 38 (3) | 19 (2) | n.d. | n.d. |
| 86 | <LOQ | < LOQ | <LOQ | < LOQ | n.d. | n.d. | n.d. | n.d. |
| 87 | <LOQ | < LOQ | n.d. | n.d. | n.d. | n.d. | n.d. | n.d. |
| 88 | n.d. | n.d. | n.d. | n.d. | 5.9 (0.2) | 3.6 (0.1) | n.d. | n.d. |
| 89 | n.d. | n.d. | <LOQ | < LOQ | n.d. | n.d. | n.d. | n.d. |
| 90 | n.d. | n.d. | <LOQ | < LOQ | n.d. | n.d. | n.d. | n.d. |
| 91 | n.d. | n.d. | n.d. | n.d. | n.d. | n.d. | n.d. | n.d. |

^a^ creatinine value not determined

DH-CIT: LOD 0.20 ng/mL urine, LOQ 0.70 ng/mL urine

DON: LOD 1.7 ng/mL urine; LOQ 5.6 ng/mL urine

DON-GlcA: LOD 1.0 ng/mL urine; LOQ 3.3 ng/mL urine

OTA: LOD: 0.010 ng/m urine L; LOQ 0.030 ng/mL urine
